# Evaluating Detection of an Inhalational Anthrax Outbreak

**DOI:** 10.3201/eid1212.060331

**Published:** 2006-12

**Authors:** David L. Buckeridge, Douglas K. Owens, Paul Switzer, John Frank, Mark A. Musen

**Affiliations:** * McGill University, Montreal, Quebec, Canada;; † Veterans Affairs Palo Alto Health Care System, Palo Alto, California, USA;; ‡ Stanford University, Stanford, California, USA;; § University of Toronto, Toronto, Ontario, Canada

**Keywords:** Syndromic surveillance, outbreak detection, simulation, evaluation, bioterrorism, research

## Abstract

Timely detection of an inhalational anthrax outbreak is critical for clinical and public health management. Syndromic surveillance has received considerable investment, but little is known about how it will perform relative to routine clinical case finding for detection of an inhalational anthrax outbreak. We conducted a simulation study to compare clinical case finding with syndromic surveillance for detection of an outbreak of inhalational anthrax. After simulated release of 1 kg of anthrax spores, the proportion of outbreaks detected first by syndromic surveillance was 0.59 at a specificity of 0.9 and 0.28 at a specificity of 0.975. The mean detection benefit of syndromic surveillance was 1.0 day at a specificity of 0.9 and 0.32 days at a specificity of 0.975. When syndromic surveillance was sufficiently sensitive to detect a substantial proportion of outbreaks before clinical case finding, it generated frequent false alarms.

## Abstract 2

One-sentence summary for table of contents: When syndromic surveillance detected a substantial proportion of outbreaks before clinical case finding, false-positive results occurred.

In the early stage of an inhalational anthrax outbreak, a 1-day delay in the initiation of chemoprophylaxis and treatment of exposed persons can result in thousands of additional deaths and millions of dollars of additional expenditures (*1**,**2*). Thus, timely detection of an inhalational anthrax outbreak is critical. Rapid detection is also important for disease outbreaks that result from other bioterrorism agents and from emerging infectious diseases, such as severe acute respiratory syndrome or avian influenza (*3*).

To detect an epidemic such as inhalational anthrax, which is nonendemic and results in severe symptoms, public health authorities have relied traditionally on identification and rapid reporting of the sentinel clinical case. However, because the perceived likelihood of a bioterrorism attack has increased, public health authorities have sought novel approaches for rapid outbreak detection. One approach that has received considerable economic investment over the past 5 years is syndromic surveillance. This approach follows prediagnostic data sources in an attempt to detect an increase in the prevalence of nonspecific symptoms. For example, the BioSense system (*4*), developed by the Centers for Disease Control and Prevention (CDC) at a cost of >$75 million (*5*), follows records of outpatient visits, pharmaceutical prescriptions, and laboratory orders in an attempt to detect disease outbreaks rapidly. Hundreds of similar systems are maintained or are under development by various groups around the world (*6*). Other examples include systems operated by the Department of Homeland Security (*5*) and academic centers in partnership with state or county public health departments (*7**–**9*).

In addition to supporting outbreak detection, these syndromic surveillance systems provide situational awareness for public health authorities and may serve other purposes. Nevertheless, a major justification for these systems is outbreak detection. Despite substantial investment in syndromic surveillance and calls for further research from groups such as the Institute of Medicine (*3*), little evidence exists to suggest how syndromic surveillance will perform relative to clinical case finding for detection of an inhalational anthrax outbreak (*10*). The reason for this lack of evidence is that data from real outbreaks are not available to evaluate the performance of syndromic surveillance alone or in comparison to clinical case finding. Moreover, even if data were available from a large-scale outbreak, those data would allow only an evaluation of performance in 1 specific setting. CDC recently endorsed the use of simulated outbreaks to address the dearth of outbreak data (*11*), but existing simulation studies have not compared detection through clinical case finding with syndromic surveillance (*12**–**14*). Our aim was to develop a model for simulating use of healthcare services after a large-scale exposure to aerosol anthrax spores and then to use this model to estimate the detection benefit of syndromic surveillance compared with clinical case finding.

## Methods

### Study Design

We developed a model to simulate the dispersion of released anthrax spores; the infection of exposed persons; the progression of disease in infected persons; and symptomatic persons' use of the healthcare system, including blood culture testing in clinical settings. Using the simulation model, we generated outbreak signals and time until the first clinical diagnosis for 3 amounts of spores released. To incorporate into the model the uncertainty in parameter values, we used a Latin hypercube sampling design, which allows many parameter values to vary simultaneously (*15*). The 3,000 simulated signals generated with this sampling strategy were superimposed in turn onto baseline administrative records of ambulatory healthcare visits in the Norfolk, Virginia, area. These records are generated daily and similar types of records are used widely for syndromic surveillance (*4**,**7**,**9*). We assessed the usefulness of syndromic surveillance by modeling the healthcare system use that would occur after an anthrax attack and superimposing this use onto actual administrative data over 1-year period. Finally, we assessed, over a range of specificity, the sensitivity and timeliness of syndromic surveillance and the detection benefit of syndromic surveillance compared with clinical case finding for each simulated outbreak. We summarize our methods in the remainder of this section and refer readers to the Technical Appendix for additional details.

### Simulation Model

**Figure 1:**
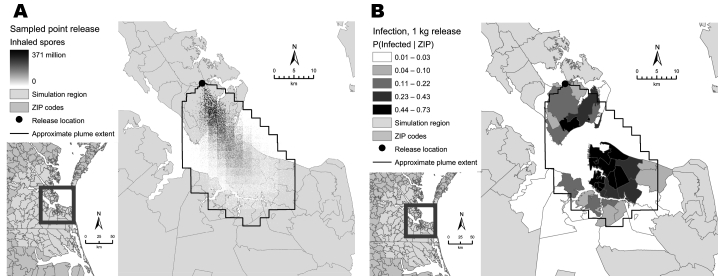
Maps showing output from dispersion (A) and infection (B) components of the simulation model. The dispersion component simulates geographic distribution of anthrax spores after an aerosol release. The infection component simulates infection of persons exposed to spores.

The simulation model builds on our previous work (*16**–**18*) and is composed of 4 components: dispersion, infection, disease, and healthcare system use. The dispersion model simulates the number of anthrax spores a person would inhale at locations throughout the region after release of aerosolized spores. We used the Hazard Prediction and Assessment Capability (HPAC) software developed by the Defense Threat Reduction Agency to simulate the dispersion of spores (*19*). The HPAC model accounts for factors such as atmospheric conditions and terrain. We simulated a point release of 3 amounts of anthrax spores: 1 kg, 0.1 kg, and 0.01 kg (Figure 1A).

The infection model simulates the number of persons infected, according to residential address and dispersion of spores (Figure 1B). The probability of infection given exposure to an amount of spores was modeled by using a probit regression model. The disease model uses a semi-Markov process to simulate the progression of infected persons through 3 discrete states of disease. Each infected person began in the incubation state and then progressed through the prodromal state and the fulminant state. The time in each state was sampled from a log normal distribution.

The healthcare use model uses a semi-Markov process to simulate the probability and timing of a symptomatic person seeking care and submission of blood for culture and culture results when care is sought. For persons in the prodromal or fulminant state of disease who sought care, the instantaneous probability of seeking care increased linearly over the duration of the state. For patients whose blood samples were cultured, the testing process was modeled as the transition through 2 discrete states: growth and isolation. The time spent in each of these states was modeled by using an exponential distribution.

### Data for Simulation Model

The infection model used an infection function corresponding to the data reported by Glassman (*20*). This is a probit model with a 50% lethal dose (LD_50_) of 8,600 spores and a slope of 0.67. Uncertainty exists about the values for many of the parameters in the disease and healthcare use models. To incorporate this uncertainty into our estimates, we used a Latin hypercube sampling approach to sample parameter values for random variables in our simulation model (*15*). This approach requires specifying equal probability bins for parameter values. We specified 3 bins for each parameter value, a narrow bin around the most likely estimate, and wider bins on either side of the estimate. Table 1 shows the bins we used for each parameter value, the probability distribution that each value parameterizes, and the sources that we used to define the bins.

**Table 1:** Sampling intervals for parameter values in the simulation model*

Parameter	Parameter value intervals		Probability distribution		Source†
Disease model					
Incubation duration, d; median	(5, 9) (9, 11) (11, 15)	Log normal		(*2**,**21**,**22*)	
Incubation duration, dispersion	(1.5, 1.9) (1.9, 2.1) (2.1, 2.5)	Log normal		(*2**,**21**,**22*)	
Prodromal duration, d; median	(1.5, 2.3) (2.3 ,2.7) (2.7, 3.5)	Log normal		(*2**,**22*)	
Prodromal duration, dispersion	(1.2, 1.4) (1.4, 1.5) (1.5, 1.7)	Log normal		(*2**,**22*)	
Healthcare use					
Probability of visit, prodromal state	(0.05, 0.25) (0.25, 0.35) (0.35, 0.55)	Bernoulli		(*23**,**24*)	
Probability of visit, fulminant state	(0.7, 0.9) (0.9, 0.95) (0.95, 1)	Bernoulli		Estimate	
Probability of respiratory syndrome, prodromal state	(0.5, 0.7) (0.7, 0.8) (0. 8,1)	Bernoulli		(*25**,**26*)	
Blood culture test, prodromal state	(0.001, 0.01) (0.01, 0.015) (0.015, 0.025)	Bernoulli		(*28*)	
Blood culture test, fulminant state	(0.7, 0.9) (0.9, 0.95) (0.95, 1)	Bernoulli		Estimate	
Sensitivity of blood culture	(0.5, 0.8) (0.8, 0.9) (0.9, 1)	Bernoulli		(*27*)	
Time until blood culture growth, d	(0.4, 0.8) (0.8, 1.0) (1.0, 1.4)	Exponential		(*30*)	
Probability of isolation given growth	(0.5, 0.8) (0.8, 0.9) (0.9, 1)	Bernoulli		(*29*)	
Time until blood culture isolation, d	(0.5, 0.6) (0.6, 0.9) (0.9, 1.5)	Exponential		(*25*)	

*Using a Latin hypercube strategy, a value for each parameter was sampled by randomly selecting 1 of the 3 intervals for the parameter and randomly sampling a value on the selected interval. The sampled values parameterize probability distributions, which are sampled for the simulation model. †References that support the parameter value intervals.

We used previous work modeling anthrax for the distribution of time periods in each disease state (*2**,**21**,**22*). For the probability of seeking care while in the prodromal disease state, cross-sectional surveys indicate that 14%–30% of persons visit a physician at some point during an episode of upper respiratory tract illness (*23**,**24*). For the fulminant disease state, we estimated the probability of seeking care before death as 90%–95%, given the severity of the symptoms in that state.

After a person made a healthcare visit, we simulated the syndrome assigned to the person by using probabilities that reflect the distribution of clinical presentations for inhalational anthrax reported in the literature (*25**,**26*). Because we considered only respiratory syndromes for surveillance, we varied directly only the probability of being assigned a respiratory syndrome to persons in the prodromal disease state.

For visits from persons in either symptomatic disease state, the estimate of sensitivity from published studies of blood culture testing was 0.8–0.9 (*27*). For a visit in the prodromal state, we estimated the probability of a physician ordering a blood culture as 0.01–0.015 on the basis of data from the National Ambulatory Medical Care Survey (*28*). For a visit in the fulminant state of disease, we estimated the probability of a blood culture test as 0.9–0.95. After gram-positive rods grew in the blood culture, we estimated the probability of isolating the organism to be 0.8–0.9 (*29*). We modeled the time until growth and isolation as exponential (*25**,**30*).

### Baseline Data and Release Scenarios

We used records of ambulatory visits in the Norfolk, Virginia, region acquired from the TRICARE health maintenance organization as a baseline onto which we superimposed simulated outbreak records. The data covered the period 2001–2003, and the simulation region included 17 clinical facilities within an ≈160-km × 200-km area that encompasses 158 ZIP codes from 2 states. Over the 3 years of available data, 427,634 persons made >5 million visits. We classified the records into syndromes by using the International Classification of Diseases, 9th Revision, Clinical Modification (ICD-9-CM) to syndrome mapping defined by the ESSENCE system (*7*) and used only 351,749 records for which persons were classified as having a respiratory syndrome. The Human Subjects Panel at the Stanford School of Medicine approved the use of these data for this study. We examined 3 scenarios defined by the amount of spores released: 1 kg, 0.1 kg, and 0.01 kg. For each scenario, we performed 1,000 simulations.

### Outbreak Detection

The time to outbreak detection through clinical case finding for a simulated outbreak was calculated for each simulated outbreak as the time between exposure to spores and the first positive blood culture. To calculate time to outbreak detection through syndromic surveillance, we superimposed the simulated records for respiratory syndrome visits onto the authentic baseline data, beginning on a randomly selected date in 2003, and then applied the outbreak detection algorithm to the combined baseline and simulated data. The outbreak detection algorithm used a time-series model (*31*) to generate daily 1-step-ahead forecasts for the total number of respiratory syndrome visits (*13*) and then applied a cumulative sum (*32*) to the forecast residual. To vary the specificity of the detection algorithm, we varied the decision threshold of the cumulative sum.

### Evaluation Metrics

To evaluate outbreak detection through syndromic surveillance, we calculated sensitivity, specificity, and timeliness at a range of decision thresholds. Timeliness is the duration between the release of anthrax spores and the first report of an outbreak. We also computed the detection benefit of syndromic surveillance relative to clinical case finding, and the proportion of runs with a detection benefit >0. Detection benefit is the potential time saved in detection from using syndromic surveillance compared with clinical case finding. The benefit is calculated as the difference in the timeliness between syndromic surveillance and clinical case finding in those simulations in which detection occurred first by syndromic surveillance. When an outbreak was not detected by syndromic surveillance, the detection benefit was 0. For a given release scenario, each of the 1,000 simulations integrated both randomness in the component model outputs as well as uncertainty in component model parameters. Each of the 1,000 simulations is a sample from the integrated distribution of possible outcomes. To indicate the spread of the integrated uncertainty distribution, we calculated the upper and lower deciles from the 1,000 simulations. For plots, we calculated 95% confidence intervals, which reflect finiteness of the simulation.

## Results

### Detection Performance of Clinical Case Finding

Because all outbreaks were ultimately detected by clinical case finding through routine blood culture, the sensitivity of this approach was 1.0 for the scenarios considered. Clinical case finding detected outbreaks from an average of 3.7 days to 4.1 days after release, with larger amounts of spores detected before smaller amounts (Table 2). Results from analyses of additional release scenarios (data not shown) suggested that the influence of amount released on time to detection was mediated, in part, through the number infected. Mean timeliness across the scenarios examined was associated with the mean number infected (Pearson's r -0.94, 95% confidence interval -0.98 to -0.79), and an increase of 10,000 infected persons resulted in a decrease in the time until detection of ≈4 hours.

**Table 2:** Average numbers of persons infected and average times to outbreak detection through clinical case finding for 3 release scenarios*

Amount released (kg)	Mean no. infected	Mean days to detection
1	49,000	3.7 (2.5, 5.0)
0.1	31,000	3.9 (2.7, 5.3)
0.01	15,000	4.1 (2.9, 5.5)

*Values in parentheses are 10th and 90th percentiles of the distribution.

### Detection Performance of Syndromic Surveillance

The sensitivity and timeliness of syndromic surveillance were influenced by the release amount and by specificity. Table 3 shows this relationship over the release scenarios examined and 2 levels of specificity. At a specificity of 0.90, a 1-kg release was detected in 100% of our simulations (sensitivity 1.0) at a mean detection time of 3.1 days. For a release that was much smaller, 0.01 kg, sensitivity was 0.94, and the mean detection time increased to 3.6 days. Although the sensitivity of syndromic surveillance was high when we set specificity to 0.90, this specificity resulted in a false alarm (false-positive detection) ≈1 every 10 days. By increasing specificity to 0.975, we reduced the false alarm rate to ≈1 every 40 days (Table 3). However, with increased specificity, the sensitivity of syndromic surveillance decreased (from 0.98 to 0.82 depending on the size of the release) and the mean time until detection lengthened to 4.3 days for a 1-kg release and to 5.1 days for a 0.01-kg release (Table 3).

**Table 3:** Sensitivity, time to outbreak detection (timeliness), proportion of outbreaks detected through syndromic surveillance before clinical case finding, and mean detection benefit of syndromic surveillance compared with clinical case finding for 3 release scenarios and 2 levels of specificity*

Amount released (kg)	Specificity 0.900 (1 false alarm every 10 d)				Specificity 0.975 (1 false alarm every 40 d)			
	Sensitivity per outbreak	Mean timeliness, d	Proportion with detection benefit	Mean detection benefit, d	Sensitivity per outbreak	Mean timeliness, d	Proportion with detection benefit	Mean detection benefit (d)
1	1.00	3.1 (0, 5)	0.59	1.0 (0, 3.3)	0.98	4.3 (2, 7)	0.28	0.32 (0, 1.0)
0.1	0.99	3.3 (0, 6)	0.55	1.0 (0, 3.5)	0.95	4.7 (2, 7)	0.24	0.33 (0, 1.1)
0.01	0.94	3.6 (0, 7)	0.51	1.1 (0, 3.7)	0.82	5.1 (2, 8)	0.19	0.33 (0, 1.3)

*Values in parentheses are 10th and 90th percentiles of the distribution.

Results from analyses of additional release scenarios (data not shown) indicated that the trends in sensitivity and timeliness across release amount were mediated to some extent by the number infected. Sensitivity was a nonlinear function of the number of persons infected, with sensitivity increasing more quickly when fewer persons were infected. At a specificity of 0.975, an increase of 10,000 infected persons resulted in a decrease in time to detection of ≈6 hours.

### Detection Benefit of Syndromic Surveillance Compared with Clinical Case Finding

**Figure 2:**
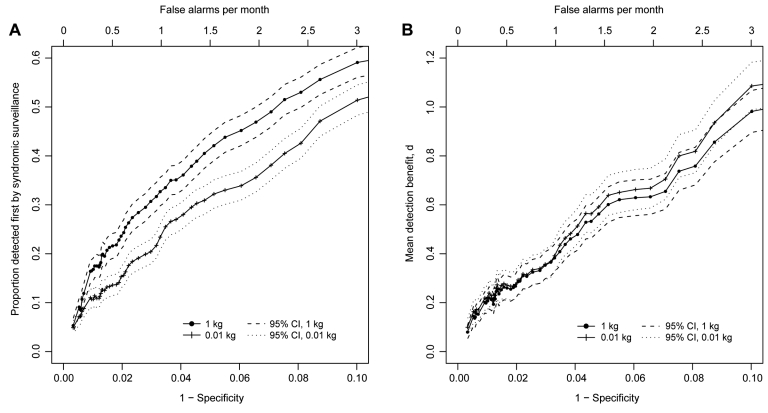
Proportion of inhalational anthrax outbreaks detected by syndromic surveillance before clinical case finding (A) and mean detection benefit of syndromic surveillance compared with clinical case finding as a function of specificity (and false-alarm rate) (B) for 3 release scenarios. CI, confidence interval.

The detection benefit of syndromic surveillance compared with clinical case finding was influenced by specificity and the release amount. Table 3 shows this relationship for the release amounts examined and 2 levels of specificity. When the specificity was 0.9, syndromic surveillance detected from 51% to 59% of outbreaks before clinical case finding, and the mean detection benefit was 1.0–1.1 days, but this specificity resulted in a false alarm every 10 days. At a specificity of 0.975, which reduced false alarms to 1 every 40 days, syndromic surveillance detected 19%–28% of outbreaks before clinical case finding and the mean detection benefit was 0.32–0.33 days, or ≈8 hours. Figure 2 shows that for the 0.01-kg and 1-kg release scenarios (results for the 0.1-kg release are similar, but are not shown), the proportion of outbreaks detected first by syndromic surveillance and the mean detection benefit of surveillance each increased as specificity decreased. Figure 2 also shows that the release amount had a strong effect on the proportion of outbreaks detected first by syndromic surveillance but that it did not have a strong effect on the mean detection benefit.

At a set specificity, syndromic surveillance tended to detect a higher proportion of outbreaks before clinical case finding with increasing release amount. The mean detection benefit, in contrast, tended to decrease when the amount of spores released increased. This decrease in average detection benefit occurred because even though syndromic surveillance detected more outbreaks before clinical case finding as the release amount increased, the detection benefit for the additional outbreaks was small, and the average detection benefit thus decreased.

## Discussion

When we compared the performance of clinical case finding with that of syndromic surveillance for detecting an inhalational anthrax outbreak, we found that clinical case finding detected outbreaks on average 3.7–4.6 days after release of spores. The ability of syndromic surveillance to detect an outbreak before clinical case finding was influenced by both specificity and release size, with specificity being the predominant factor. Our results suggest that syndromic surveillance could detect an inhalational anthrax outbreak before clinical case finding. However, we regularly observed a detection benefit only when syndromic surveillance operated at a specificity in the range of 0.9, which corresponds to 1 false alarm every 10 days. When operating at this relatively low specificity with a concomitant high sensitivity, syndromic surveillance detected outbreaks, on average, 1 day before clinical case finding did.

One of the most useful findings of our study was the tradeoff between sensitivity and specificity of syndromic surveillance. To reduce the false alarm rate, specificity must be high. However, as specificity increased in our study, the sensitivity of syndromic surveillance decreased, and the proportion of outbreaks that was detected first by syndromic surveillance decreased more substantially. If the response to a result from syndromic surveillance is resource intensive and includes follow-up investigations in multiple healthcare settings, then a false alarm rate of 1 every 10 days may be too high for such a system to be useful. Alternatively, if public health personnel can rule out false-positive results with minimal investment, then a higher rate of false alarms may be acceptable.

The detection benefit of syndromic surveillance might be an important lead, depending on the action triggered by a surveillance alarm. Because many clinical and public health departments have defined protocols for actions after clinical confirmation of an inhalational anthrax case (*33*), the action after detection of a clinical case is fairly well defined in many jurisdictions. In contrast, the appropriate action after a result from syndromic surveillance system is not well-defined (*34*). For example, some public health departments routinely wait 1 day for a second alarm before taking action (*35*). This strategy could eliminate the potential detection benefit of syndromic surveillance. Another concern is the relatively low specificity at which syndromic surveillance must operate to consistently result in a detection benefit. A system producing this many false alarms may result in excessive costs, and users may minimize the importance of these results.

To be useful, however, syndromic surveillance does not necessarily have to detect all outbreaks, or even most outbreaks, before a clinician detects the first case. The additional lead in detection offered by syndromic surveillance in some outbreaks may result in enough benefit to support the use of syndromic surveillance. Syndromic surveillance may also be useful for applications other than detecting an outbreak caused by bioterrorism; e.g., for detecting other types of disease outbreaks (*36*), for ruling out the existence of an outbreak, or for evaluating the effect of a public health intervention. Assessment of the question of the utility of syndromic surveillance in general would require consideration of a broader range of costs and benefits than we included in our study.

Our methods are an advance over those used in previous studies because we were able to examine rigorously, within a single modeling framework, the ability of clinical case finding and syndromic surveillance to detect anthrax outbreaks. The nature of our model allowed us to vary some outbreak characteristics directly (e.g., release amount) and to incorporate the uncertainty in parameter values into our final estimates of detection performance and detection benefit. Although our sampling approach did allow us to vary many parameter values simultaneously, it did not clarify how the results vary in relation to changes in the value of a single parameter. Our estimate of detection performance through syndromic surveillance is comparable to estimates observed through studies that used simulation models (*12**,**37*), but those studies did not allow direct comparison of detection through syndromic surveillance with detection through clinical case finding. Our estimate of the time until detection through clinical case finding is longer than the estimate used by the authors of a study aimed at modeling response strategies to an anthrax outbreak (*2*), but those authors did not provide a clear rationale for the value they chose. An initial presumptive diagnosis may occur earlier than the first positive blood culture result (e.g., through clinical symptoms and chest radiographs), but a decision for large-scale intervention would likely not be made until at least after the first definitive diagnosis was made.

In our study, we considered 1 approach to syndromic surveillance for an outbreak resulting from 1 type of organism, and we considered clinical case finding through 1 type of routinely applied diagnostic test. There are many different approaches to syndromic surveillance; e.g., different types of data and different detection algorithms. Although different approaches to surveillance might produce different results, the choice of the infectious organism is likely to have a greater effect on results. Anthrax is relatively unique among bioterrorism agents in that a routinely used diagnostic test (i.e., blood culture) will identify the organism definitively. The benefit of syndromic surveillance relative to clinical case finding may therefore be greater for outbreaks caused by other organisms, and an anthrax outbreak may be a worst-case scenario for syndromic surveillance.

Syndromic surveillance detected an inhalation anthrax outbreak before the first clinical case was diagnosed in as many as half of simulated outbreaks. However, the potential detection benefit of syndromic surveillance compared with clinical case finding depended critically on the specificity and sensitivity at which a surveillance system operated and on the size of the outbreak. When syndromic surveillance was sufficiently sensitive to detect a substantial proportion of outbreaks, it generated frequent false alarms. Public health authorities should be aware that the potential detection benefit of syndromic surveillance compared with clinical case finding is influenced strongly by the specificity at which a surveillance system operates. To help detect outbreaks more rapidly, future research should examine the cost-effectiveness of syndromic surveillance and explore approaches to linking syndromic surveillance and clinical case finding more closely.

## Supplementary Material

Technical Appendix Additional information about the methods used in the simulation study.
