# Supplementary material for: Evaluating Detection of an Inhalational Anthrax Outbreak
Source: Emerg Infect Dis. 2006 Dec;12(12):1942–9. doi: 10.3201/eid1212.060331 (PMC3291344; doi:10.3201/eid1212.060331)
Supplement: Technical Appendix — Additional information about the methods used in the simulation study. [file 06-0331-Techapp_13p.pdf]

This material, provided by the author(s) as a supplement to Evaluating Detection of an Inhalational Anthrax Outbreak, is not part of Emerging Infectious Diseases contents.

Technical Appendix

In this document we provide additional information about the methods used in the simulation study that we describe in the main paper. In section 1 we describe the simulation model and in section 2 we describe the study design.

1 Simulation Model

The simulation model used in this study builds on our earlier work [5–7].

1.1 Dispersion

The dispersion model takes as input the amount of anthrax spores released and returns as output the number of spores an individual would inhale at locations on a regular grid overlaid upon the study region. To model the dispersion of anthrax spores, we used the Hazard Prediction and Assessment Capability (HPAC) software, which is developed by the US Defence Threat Reduction Agency (DTRA) to allow modeling of threats from weapons of mass destruction [8]. This software uses meteorological and terrain data to interpolate time-integrated exposure concentrations from a release scenario onto a spatial grid, relying on the second-order cluster integrated puff model [24].

The HPAC software outputs a mean exposure plume for a release scenario. The mean plume is determined within HPAC by simulating many individual plumes and then calculating the mean and standard deviation of exposure at each location on an exposure grid. We did not use the mean plume directly because it smooths out the variation in spore concentration within a plume and because the mean plume tends to overestimate exposure at the margins of the plume. To address these limitations of the mean plume, we re-sampled randomly from the mean plume.

The release scenario we used for all release amounts was a stationary release in the area of the Langley Air Force Base (37.1N, 76.4W), using weather data for Veteran's Day (November 11, 5 am EST, 2 m s^{-1} NNW wind, clear skies), with a 2 m release height, and a 10 s duration. The HPAC software was set to calculate a boundary layer and large-scale variability, and to include terrain effects. We assumed that 1 kg of spores contained 10^{15} spores and we modeled the dispersion of 1 kg, 0.1 kg, and 0.01 kg of spores. We calculated a mean plume at each release amount and then re-sampled 1,000 random plumes from each mean plume. The HPAC model provides the time-integrated concentration

This material, provided by the author(s) as a supplement to Evaluating Detection of an Inhalational Anthrax Outbreak, is not part of Emerging Infectious Diseases contents.

at each location and we assumed that individuals breathed at a rate of 0.0005 m^3s^{-1} to determine the number of spores an individual would inhale at locations throughout the study region.

1.2 Infection

The infection model takes as input the number of spores an individual would inhale at locations on a regular grid (output from the dispersion model) and the population by ZIP code. The model returns the number of infected individuals by residential ZIP code. We assumed that all individuals were exposed at their home address and that each individual had a uniform probability of being exposed at any location within their home ZIP code. To determine the probability of infection following exposure to a given number of spores, we used a function corresponding to the data reported by Glassman following experimental exposure of primates [10]. This is a probit model with an LD50 of 8,600 spores and a slope of 0.67. Figure 1 shows a plot of the infection function. This function allows for infection at low dose exposure. For example, approximately 2.5% of individuals exposed to 10 spores will become infected.

To determine the number infected by home ZIP code, we first determined the probability of infection for each ZIP code Z_i as the average probability of infection across the ZIP code:

$$p_{Z_i} = \frac{1}{n_i}\sum_{j=1}^{n_i} f(c_j),$$

where there are n_i cells from the exposure grid within ZIP code Z_i and $f(c_j)$ is the infection function that returns the probability of infection given the number of spores inhaled in cell c_j. The number of individual infected within a ZIP code I_{Z_i} was then sampled from a binomial distribution:

$$I_{Z_i} \sim binomial(N_{Z_i}, p_{Z_i}),$$

where N_{Z_i} is the population of ZIP code Z_i.

1.3 Disease

The disease model takes as input the number of infected individuals and returns a disease path for each individual. The disease path describes the amount of time spent in each discrete disease state. We used a semi-Markov process to model progression of an individual with inhalational anthrax through three disease states: incubation, prodromal, and fulminant (Figure 2) [26].

The definition of the semi-Markov process requires identification of the states, including the holding-time functions, and specification of the transition probabilities between states. The initial state in the model was incubation, followed by certain transition to the prodromal state, and then the fulminant state. For holding-time functions, we used the lognormal distribution, which appears to describe the duration of incubation for many diseases [20, 23], including inhalation

This material, provided by the author(s) as a supplement to Evaluating Detection of an Inhalational Anthrax Outbreak, is not part of Emerging Infectious Diseases contents.

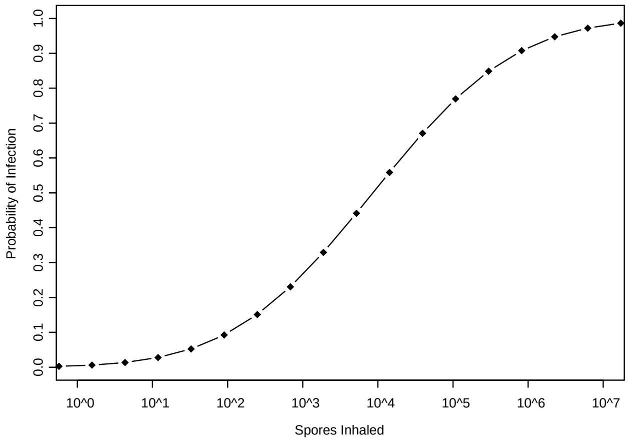

Figure 1: The infection function for inhaled anthrax determined mainly from primate research [10].

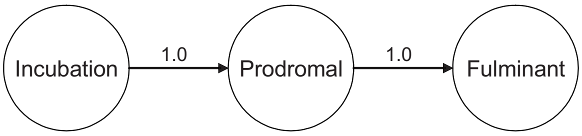

Figure 2: The disease model for inhalational anthrax. Infected individuals all pass through three disease states. In the incubation state individuals have no symptoms. In the prodromal state individuals experience an influenza-like illness. Finally, in the fulminant state individuals experience severe symptoms, such as shock. The holding-time function in each state is a lognormal distribution with parameters shown in Table 1.

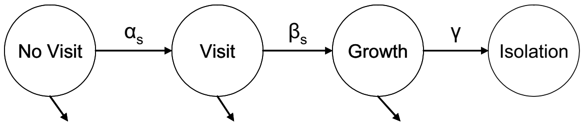

Figure 3: The health-care utilization model. When individuals enter the prodromal or fulminant disease state, they enter the health-care utilization model. The probability of making a visit (α_s) varies by disease state (Table 1). The probability of a positive blood culture (β_s) is the product of the probability of ordering a test (which varies by disease state) and the sensitivity of the test (which does not vary by disease state). The probability of isolating the organism does not vary by disease state. The holding-time function for the 'No Visit' state is triangular, with a duration equivalent to the length of the disease state. Holding-time functions for the 'Visit' and 'Growth' states are exponential, with parameters shown in Table 1.

anthrax [4, 17]. The values used to parameterize the holding-time functions are taken from observational studies of human exposure [4, 17] and other modeling studies [25, 26], and are shown in Table 1.

1.4 Health-Care Utilization

The health-care utilization model takes as input a set of disease paths and for each path performs three tasks: (1) it identifies if and when individuals seek care in each disease state, (2) it determines the presenting syndrome for individuals that seek care, and (3) it identifies the timing and results of blood culture testing once care is sought.

We used a semi-Markov process to model health-care utilization (Figure 3). A separate process was used to describe health-care utilization in each of the prodromal and fulminant disease states. Both processes had the same states and transitions (Figure 3), but some values for holding-time functions and transition probabilities differed between the disease states (i.e., those states with a s subscript in Figure 3) and the values used in the simulation study are shown in Table 1.

The transition from 'No Visit' to 'Visit' represents an individual seeking care at an ambulatory clinic or emergency department. The probability of this transition occurring (α_s) differs between disease states (s). We set the probability of a visit in the prodromal disease state (α_p), to approximately 0.30 because cross-sectional surveys suggest that this proportion of individuals visit a physician at some point during an episode of upper respiratory tract illness [15, 18]. For the fulminant disease state (α_f), we estimated the probability of seeking care as approximately 95% given the severity of the symptoms in that state.

The transition from 'Visit' to 'Growth' represents an individual having a positive blood culture test after making a visit. The probability of this transition (β_s) is the product of the probability of performing a blood culture test (β_s^1) and

This material, provided by the author(s) as a supplement to Evaluating Detection of an Inhalational Anthrax Outbreak, is not part of Emerging Infectious Diseases contents.

the sensitivity of the test (β^2, i.e., $\beta_s = \beta_s^1 \times \beta^2$). The probability of performing a test in the prodromal state (β_p^1), was estimated from the National Ambulatory Health Care Survey as approximately 0.0125 [9]. In the fulminant state, we assumed that the probability of a blood culture test (β_f^1) was approximately 0.95. We relied on published studies of blood-culture testing to estimate the sensitivity of blood-culture testing in both symptomatic disease states (β^2) as approximately 0.85 [21].

The final transition, from 'Growth' to 'Isolation', represents the decision to isolate the organism from a blood culture bottle growing gram-positive rods. We relied on data from a recent survey to estimate this value (γ) as approximately 0.9 [2].

In addition to a transition probability, each of the first three states in the health-care utilization model also requires a holding-time function. The holding-time function for the 'No Visit' state models the distribution of time to seeking care, given that care is sought. We used a right triangular distribution fit to the time spent in the disease state. So, for example, if an individual had a prodromal disease state duration of 10 days, then the probability of seeking care at the instant of entering the disease state would be zero, and the instantaneous probability of seeking care would increase linearly to 0.2 at ten days, with a mean time to seeking care of 6.7 days.[1] This approach to modeling visits effectively limits individuals to a single visit in each disease state. The selection of a triangular distribution reflects the lack of published evidence about the timing of health-care utilization following the onset of symptoms.

The holding time function for 'Visit' reflects the distribution of times until growth occurs given that the test is positive. The holding time function for 'Growth' is the distribution of times until the organism is isolated given than a decision is made to isolate a specific organism. We modeled both these holding times as exponential with means obtained from published reviews of blood-culture testing [1, 11].

Finally, for individuals that made a health care visit, we simulated the syndrome assigned to the individual using probabilities that reflect the distribution of clinical presentations for inhalational anthrax reported in the literature [11, 12]. As we consider only respiratory syndromes for surveillance, we modeled only the probability of being assigned a respiratory syndrome in the prodromal disease state, which we estimated at approximately 0.75 (Table 1).

2 Study Design

2.1 Generation of Simulated Signals

We generated 1,000 simulated outbreak signals at each release amount for a total of 3,000 signals. To generate the simulated outbreaks for each release amount,

[1] These values result from the properties of the triangular distribution, which is defined by three parameters: a, b, and c. In a right triangular distribution, b = c. The maximum point density is 2 / (b - a) and the mean is (a + b + c) / 3.

This material, provided by the author(s) as a supplement to Evaluating Detection of an Inhalational Anthrax Outbreak, is not part of Emerging Infectious Diseases contents.

we first sampled an exposure grid and calculated the number infected by home ZIP code. The next step was to select a set of disease and health-care utilization parameters. We then generated a disease path for each infected individual, the timing of visits to physicians for symptomatic individuals, the administrative codes generated through visits, and the occurrence, timing and results of blood culture testing.

Due to the large number of parameter values in the disease and health-care utilization components of the simulation model, we used Latin hypercube sampling (LHS) to select parameter values for each simulation run of these components. LHS is an approach to sampling parameter values from a high-dimensional parameter space in order to obtain estimates of output variables that are more efficient and precise than would be obtained with simple random sampling [16]. When specifying a simulation model there are K parameters. A given run of the simulation model requires a value for each parameter, or a set of parameter values $\mathbf{X} = \{X_1, \ldots, X_K\}$. Each parameter has a space of possible values $\mathbf{S} = \{S_1, \ldots, S_K\}$. In LHS the space for each parameter is partitioned into N intervals of probability size $1/N$. The Cartesian product of these intervals partitions S into N^K cells, which form a hypercube. To obtain $\mathbf{X}$ for a simulation run requires randomly sampling a partition for each parameter, and then sampling a parameter value from within that partition, assuming that values are uniformly distributed within a partition. Table 1 shows the parameter value intervals used in the simulation study.

In order to reduce the variance between scenarios, we used the same 1,000 sets of disease and health-care utilization parameters, selected through LHS, for each scenario. In other words, the first runs for each of the 3 release amounts all used the first set of sampled parameters, the second runs all used the second set of sampled parameters, and so on. Similarly, we sampled 1,000 exposure grids and all 3 scenarios used the same 1,000 re-sample exposure plumes.[2] Finally, the same random number generator with the same seed value was used for each scenario. We used a combined multiple recursive generator as proposed and implemented by L'Ecuyer with the default initial seed [14]. This sampling strategy was intended to improve the efficiency of the simulation and reduce the variance of the output variables [13]. The net result is to facilitate comparison of the results across the different scenarios.

2.2 Combination of Simulated Data with Baseline Data

We first defined a 330 day interval on the baseline data from January 19, 2003 until December 15, 2003 as possible starting dates for a simulated outbreak. The gap at the beginning of the year was to allow a period for the detection algorithm to initialize using test data, and the gap at the end of the year was to ensure that injected outbreaks did not run off the end of the test data. We then selected 1,000 random dates for injecting outbreaks. Each of the 330 dates

[2]The pattern of dispersion is constant and the number of spores scales linearly with amount release. We therefore simulated a 1 kg release and scaled the results for the two other release amounts.

This material, provided by the author(s) as a supplement to Evaluating Detection of an Inhalational Anthrax Outbreak, is not part of Emerging Infectious Diseases contents.

Parameter	Distribution	Parameter Value Intervals	Source
**Holding-Time Functions**			
Disease Model			
Incubation (days), median	lognormal	[5, 9] [9, 11] [11, 15]	[4]
Incubation, dispersion∗	lognormal	[1.5, 1.9] [1.9, 2.1] [2.1, 2.5]	[4]
Prodromal (days), median	lognormal	[1.5, 2.3] [2.3, 2.7] [2.7, 3.5]	[26]
Prodromal, dispersion	lognormal	[1.2, 1.4] [1.4, 1.5] [1.5, 1.7]	[26]
Fulminant (days), median†	lognormal	[1.5]	[26]
Fulminant dispersion†	lognormal	[1.44]	[26]
Health-Care Utilization Model			
Time until visit	right triangular	disease-state duration‡	Estimate
Time until blood culture growth (days)	exponential	[0.4, 0.8] [0.8, 1.0] [1.0, 1.4]	[1]
Time until blood culture isolation (days)	exponential	[0.5, 0.6] [0.6, 0.9] [0.9, 1.5]	[11]
**Transition Probabilities**			
No Visit to Visit (α_s)			
Prodromal state visit (α_p)	Bernoulli	[0.05, 0.25] [0.25, 0.35] [0.35, 0.55]	[18]
Fulminant state visit (α_f)	Bernoulli	[0.7, 0.9] [0.9, 0.95] [0.95, 1]	Estimate
Visit to Growth ($\beta_s = \beta_s^1 \times \beta^2$)			
Blood culture test, prodromal state (β_p^1)	Bernoulli	[0.001, 0.01] [0.01, 0.015] [0.015, 0.025]	[9]
Blood culture test, fulminant state (β_f^1)	Bernoulli	[0.7, 0.9] [0.9, 0.95] [0.95, 1]	Estimate
Sensitivity of blood culture (β^2)	Bernoulli	[0.5, 0.8] [0.8, 0.9] [0.9, 1]	[21]
Growth to Isolation (γ)			
Prodromal and fulminant states	Bernoulli	[0.5, 0.8] [0.8, 0.9] [0.9, 1]	Estimate
**Assignment of Presenting Syndrome**			
Probability of respiratory syndrome, prodromal state	Bernoulli	[0.5, 0.7] [0.7, 0.8] [0.8, 1]	[12]

Table 1: Ranges of parameter values used in the Latin hypercube sampling. The indicated source was used to identify the best estimate of the parameter value interval and inform the range of the intervals. ∗Following Sartwell [23], the parameter $d = e^s$, where s^2 is the variance, is referred to as the *dispersion factor*. †We did not model uncertainty about the fulminant disease state because the there is general agreement that this state has a short duration. ‡The length of disease state was used to parameterize the triangular visit-time distribution.

This material, provided by the author(s) as a supplement to Evaluating Detection of an Inhalational Anthrax Outbreak, is not part of Emerging Infectious Diseases contents.

was used 3 times and ten dates were sampled randomly from the 330 dates to be used a fourth time. All 100 dates were then shuffled randomly.

To inject the simulated outbreak signals for a release amount, we used the following method. Each release amount had set of simulated outbreaks, $O = \{O_1, \ldots, O_{1000}\}$, and the set of randomly ordered dates, $D = \{D_1, \ldots, D_{1000}\}$. For outbreak j, $j \in \{1, \ldots, 1000\}$, we selected O_j and D_j. The outbreak O_j is a time series of counts, lasting n days and representing the visits for respiratory conditions from the day of the simulated release until the day of the peak incidence of cases.

The outbreaks series O_j is an ordered set of values, $O_j = \{o(1), \ldots, o(n)\}$, which we define to run from D_j until $D_j + n - 1$, or $O_j = \{o(D_j), \ldots, o(D_j + n - 1)\}$. The background time series is also an ordered set of values, $B = \{b(1), \ldots, b(m)\}$. For inject j, we extract a subset of the background time series $B_j = \{b(D_j - g), \ldots, b(D_j), \ldots, b(D_j + n - 1)\}$, where g is the length of the lead-in gap, which is the amount of time in the inject series before the beginning of the outbreak. We then define the inject series $I_j = \{i(D_j - g), \ldots, i(D_j + n - 1)\}$, with the entries in the series defined as,

$$i(k) = \begin{cases} b(k) + o(k) & \text{if } k \geq D_j \text{ and } k < D_j + n \\ b(k) & \text{otherwise} \end{cases}$$

In other words, the inject series is formed by adding the values of the outbreak series to the values of the background series, after aligning the two series so that the first day of the outbreak series is added to day D_j in the background series. We then applied the outbreak detection algorithm to each day in the inject series to generate alarm values from day $D_j - g$ to day $D_j + n - 1$. The lead-in gap $g = 16 + (3 \times 28) = 100$ days, and the first 16 values were excluded from the alarm series as they were required to initialize the temporal forecast algorithm.[3] This left an 84 day (approximately 3 months) initilization period.

2.3 Outbreak Detection Algorithm

We used an autoregressive seasonal integrated moving average (SARIMA) model [3] to calculate one-step-ahead daily forecasts of respiratory syndrome counts and then used a cumulative sum [19] to detect positive deviations in the forecast residuals. Other researchers have used a similar approach to outbreak detection in a surveillance setting [27].

To fit the SARIMA model, we used two years of data for respiratory syndrome visit counts (2001 to 2002, inclusive) and followed a procedure similar to that described elsewhere [22]. This entailed subtracting the overall mean, day-of-week means, month means, and holiday means from the original count data to give a series centered on zero. We used trimmed means (alpha = 0.1) for both day-of-week and month to minimize the influence of outliers. We then assessed the temporal autocorrelation in this series and fit a SARIMA model using a standard approach to model specification [3].

[3]The number of days excluded is determined by the number and order or terms in the time-series model

This material, provided by the author(s) as a supplement to Evaluating Detection of an Inhalational Anthrax Outbreak, is not part of Emerging Infectious Diseases contents.

We evaluated the fit of the SARIMA model to the training (2001 to 2002) and test data (2003) using the mean absolute percentage error (MAPE), defined as

$$MAPE = \frac{1}{j}\sum_{j=1}^{m}\frac{|\mu_j - x_j|}{x_j},$$

where there are m days in the training interval, μ_j is the forecast value on day j and x_j is the observed value. After subtracting the overall mean and means for day-of-week, month and holiday, the zero-centered series exhibited temporal autocorrelation at short lags on the order of days, and cyclical lags of order seven. We found that a SARIMA model (2,0,1) x (2,0,1)7 had the best fit to the zero-centered series. One-step-ahead forecasts from the SARIMA model resulted in a mean absolute percentage (MAPE) of 14.9% on the training data, which implies that the forecast values were, on average, within 14.9% of the true value. This fit is similar to or better than the fit reported by others using the same algorithm and similar data [22].

To detect temporal aberrancies in the observed counts, we applied a cumulative sum to the standardized forecast residual and declared an aberrancy when the cumulative sum exceeded a threshold. We calculated the standardized residual for each day as the observed total respiratory visit count minus the one-step-ahead forecast from the SARIMA model, divided by the standard error of the forecast. Standardization, or dividing the residual by the standard error of the forecast, resulted in residuals with a standard normal distribution.

We then applied a one-sided cumulative sum to the standardized residuals to detect a positive shift in the mean of the residual series:

$$S_t = max(0, S_{t-1} + ((X_t - (\mu_0 + k\sigma_x))/\sigma_x)).$$

The cumulative sum requires four parameters: the series mean μ_0, the series standard deviation σ_x, the shift k, and the decision threshold h. The shift specifies, in standard deviations, the minimum detectable change in the mean. We set the shift at 0.5 standard deviations and declared an aberrancy for a given day, j, when the cumulative sum crossed the decision threshold, or

$$A(h)_j = \begin{cases} 1 & \text{if } S_j > h \\ 0 & \text{otherwise.} \end{cases}$$

We varied h between 0 and 10 to determine the outbreak detection performance over a range of thresholds.

2.4 Evaluation of Outbreak Detection Performance

2.4.1 Sensitivity

Sensitivity is the probability of an alarm given an outbreak, or

$$Sensitivity = P(A|O) = \frac{n(A,O)}{n(O)},$$

This material, provided by the author(s) as a supplement to Evaluating Detection of an Inhalational Anthrax Outbreak, is not part of Emerging Infectious Diseases contents.

where $n(O)$ is the number of outbreaks and $n(A, O)$ is the number of outbreaks during which at least one alarm sounded. We calculated sensitivity for detecting an outbreak following a given release amount at a decision threshold h as:

$$Se(h) = \frac{1}{n}\sum_{i=1}^{n} \min(1, \sum_{j=1}^{m_i} A(h)_{ij}),$$

where there are n simulation runs and simulated outbreak i has m_i days from onset to peak incidence. Note that sensitivity is computed using only the outbreak intervals and no distinction is made as to when in the course of an outbreak alarms occur.

2.4.2 Specificity

Specificity is the probability of no alarm given that there is no outbreak, or

$$Specificity = P(\overline{A}|\overline{O}) = \frac{n(\overline{A}, \overline{O})}{n(\overline{O})},$$

where $n(\overline{O})$ is the number of days in the test data and $n(\overline{A}, \overline{O})$ is the number of alarms when the algorithm is applied to the test data without any superimposed outbreaks. We calculated specificity at a decision threshold h as:

$$Sp(h) = \frac{1}{m}\sum_{j=1}^{m} A(h)_j,$$

where there are m days in the test data set. Note that specificity is calculated using only non-outbreak data. We therefore assume that any alarm in the test data without a superimposed outbreak is a false alarm.

2.4.3 Timeliness

Timeliness is the time between the onset of the outbreak and the first alarm sounded during an outbreak. We calculated timeliness for a single simulated outbreak as:

$$T(h) = \min_j (j : A(h)_i = 1),$$

where there are m_i days from the onset until the peak of an outbreak i and timeliness is not defined if $\sum_{j=1}^{m_i} A(h)_j = 0$. Note that the mean timeliness for a set of outbreaks is not necessarily monotonically increasing or decreasing with the threshold. The reason for this potential variation in timeliness is that as the thresholds changes, new outbreaks may be detected at the changed threshold. The mean is re-calculated incorporating the timeliness from these additional outbreaks, but there is no guarantee that the timeliness for these new outbreak will be less than (or greater than) the mean timeliness before their inclusion.

The effect of newly incorporated outbreaks is less at lower specificity, where there are already a considerable number of outbreaks detected. At higher specificity, though, the derivative of the mean timeliness can change from negative to positive as the threshold changes.

Timeliness for detection through clinical case-finding in a simulation run was calculated as the minimum of the times to a positive blood culture diagnosis for all positive blood cultures ordered for visits occurring in either disease state which were assigned any syndrome.

2.4.4 Detection Benefit

Detection benefit is the potential gain in time to detection from using a new detection method relative to a standard or existing method. For a new detection method A and an existing method B, the benefit of A over B for a single outbreak is calculated as the difference in the timeliness using the two methods, or

$$D_{AB}(h) = \max(0, T_B(h) - T_A(h)).$$

The detection benefit is always greater than or equal to zero. Note that, as with timeliness, the mean detection benefit is not necessarily monotonically increasing or decreasing with the threshold.

Another measure of detection benefit is the proportion of times that method A detects an outbreak before method B. For a single outbreak, this is equivalent to calculating a binary measure in place of the continuous detection benefit, or

$$P_{AB}(h) = \begin{cases} 0 & \text{if } D_{AB}(h) = 0 \\ 1 & \text{if } D_{AB}(h) > 0. \end{cases}$$

References

[1] SE Beekmann, DJ Diekema, KC Chapin, and GV Doern. Effects of rapid detection of bloodstream infections on length of hospitalization and hospital charges. *Journal of Clinical Microbiology*, 41(7):3119–25, 2003.

[2] EM Begier. Gram-positive Rod Surveillance for Early Anthrax Detection. *Emerg Infect Dis*, 11(9):1483–1486, Sep 2005.

[3] GP Box and GM Jenkins. *Time Series Analysis, Forecasting and Control.* Prentice Hall, Englewood Cliffs, Revised edition, 1976.

[4] R Brookmeyer, N Blades, M Hugh-Jones, and DA Henderson. The statistical analysis of truncated data: application to the Sverdlovsk anthrax outbreak. *Biostatistics*, 2(2):233–247, 2001.

[5] DL Buckeridge. *A Method for Evaluating Outbreak Detection in Public Health Surveillance Systems that Use Adminstrative Data.* PhD thesis, Stanford University, 2005.

This material, provided by the author(s) as a supplement to Evaluating Detection of an Inhalational Anthrax Outbreak, is not part of Emerging Infectious Diseases contents.

[6] DL Buckeridge, H Burkom, A Moore, J Pavlin, P Cutchis, and W Hogan. Evaluation of syndromic surveillance systems–design of an epidemic simulation model. *MMWR Morb Mortal Wkly Rep*, 53 Suppl:137–43, 2004.

[7] DL Buckeridge, P Switzer, D Owens, D Siegrist, J Pavlin, and M Musen. An evaluation model for syndromic surveillance: assessing the performance of a temporal algorithm. *MMWR Morb Mortal Wkly Rep*, 54 Suppl:109–15, 2005. 1545-861X (Electronic) Journal Article.

[8] The Defense Threat Reduction Agency. *The HPAC User's Guide. Hazard Prediction and Assessment Capability, Version 4.0*, 2001.

[9] National Center for Health Statistics. Public use data tapes: National ambulatory medical care survey 2003. Technical report, National Center for Health Statistics, 2003.

[10] HN Glassman. Industrial inhalation anthrax, discussion. *Bacteriological Review*, 30:657–659, 1965.

[11] TV Inglesby, T O'Toole, DA Henderson, JG Bartlett, MS Ascher, E Eitzen, AM Friedlander, J Gerberding, J Hauer, J Hughes, J McDade, MT Osterholm, G Parker, T M Perl, P K Russell, and K Tonat. Anthrax as a biological weapon, 2002: updated recommendations for management. *Journal of the American Medical Association*, 287(17):2236–52, 2002.

[12] JA Jernigan, DS Stephens, DA Ashford, C Omenaca, MS Topiel, M Galbraith, M Tapper, TL Fisk, S Zaki, T Popovic, RF Meyer, CP Quinn, SA Harper, SK Fridkin, JJ Sejvar, CW Shepard, M McConnell, J Guarner, WJ Shieh, JM Malecki, JL Gerberding, JM Hughes, BA Perkins, and Anthrax Bioterrorism Investigation Team. Bioterrorism-related inhalational anthrax: the first 10 cases reported in the United States. *Emerging Infectious Diseases*, 7(6):933–44, 2001.

[13] AM Law and WD Kelton. *Simulation modeling and analysis.* McGraw-Hill, Third edition, 2000.

[14] P L'Ecuyer. Random number generation. In JE Gentle, E Härdle, and Y Mori, editors, *Handbook of Computational Statistics*, pages 35–70. Springer-Verlag, 2004.

[15] WJ McIsaac, N Levine, and V Goel. Visits by adults to family physicians for the common cold. *The Journal of Family Practice*, 47(5):366–9, 1998.

[16] MD McKay, RJ Beckman, and WJ Conover. A comparison of three methods for selecting values of input variables in the analysis of output from a computer code. *Technometrics*, 21:239–245, 1979.

[17] M Meselson, J Guillemin, M Hugh-Jones, A Langmuir, I Popova, A Shelokov, and O Yampolskaya. The Sverdlovsk anthrax outbreak of 1979. *Science*, 266(5188):1202–8, 1994.

This material, provided by the author(s) as a supplement to Evaluating Detection of an Inhalational Anthrax Outbreak, is not part of Emerging Infectious Diseases contents.

[18] KB Metzger, A Hajat, M Crawford, and F Mostashari. How many illnesses does one emergency department visit represent? Using a population-based telephone survey to estimate the syndromic multiplier. *MMWR Morbidity and Mortality Weekly Report*, 53 Suppl:106–11, 2004.

[19] E Page. Continuous inspection schemes. *Biometrika*, 41:100–115, 1954.

[20] P Philippe. Sartwell's incubation period model revisited in the light of dynamic modeling. *Journal of Clinical Epidemiology*, 47(4):419–33, 1994.

[21] LG Reimer, ML Wilson, and MP Weinstein. Update on detection of bacteremia and fungemia. *Clinical Microbiology Reviews*, 10(3):444–65, 1997.

[22] BY Reis, M Pagano, and KD Mandl. Using temporal context to improve biosurveillance. *Proceedings of the National Academy of Sciences USA*, 100(4):1961–5, 2003.

[23] PE Sartwell. The distribution of incubation periods of infectious diseases. *American Journal of Hygiene*, 51:310–318, 1950.

[24] RI Sykes, SF Parker, DS Henn, and WS Lewellen. Numerical simulation of ANATEX tracer data using a turbulence closure model for long-range dispersion. *Journal of Applied Meteorology*, 32:929–947, 1993.

[25] GF Webb and MJ Blaser. Mailborne transmission of anthrax: Modeling and implications. *Proceedings of the National Academy of Sciences USA*, 99(10):7027–32, 2002.

[26] LM Wein, DL Craft, and EH Kaplan. Emergency response to an anthrax attack. *Proceedings of the National Academy of Sciences USA*, 100(7):4346–4351, 2003.

[27] GD Williamson and HG Weatherby. A monitoring system for detecting aberrations in public health surveillance reports. *Statistics in Medicine*, 18(23):3283–98, 1999.
